# The Electronic Nose’s Emerging Role in Respiratory Medicine

**DOI:** 10.3390/s18093029

**Published:** 2018-09-10

**Authors:** Roberto Gasparri, Giulia Sedda, Lorenzo Spaggiari

**Affiliations:** 1 Department of Thoracic Surgery, IEO, European Institute of Oncology IRCCS, 20141 Milan, Italy; giulia.sedda@ieo.it (G.S.); lorenzo.spaggiari@ieo.it (L.S.); 2 Department of Oncology and Hemato-oncology, University of Milan, 20122 Milan, Italy

**Keywords:** COPD: electronic nose, clinical research, sensors, volatile organic compounds, lung cancer

## Abstract

New interest has grown in the respiratory disorder diagnosis and monitoring, throughout electronic nose technologies. This technology has several advantages compared to classic approach. In this short letter, we aim to emphasize electronic nose role in respiratory medicine.

In the last Chronic Obstructive Pulmonary Disease (COPD) International Conference in Birmingham, a work of Van Geffen and colleagues [1] that suggests the possibility of monitoring COPD using e-noses was presented.

E-noses are artificial, small and portable devices composed of sensors, which are able to respond to compounds’ concentration pattern variation. This technology has grown in importance in the medical field only in the last years, but it has been used in in food analysis, environmental monitoring and for military purposes for decades [2]. Classically in clinical practice inertial sensors were used, able to capture outcomes in observational applications, such as electrodes for cardiac rhythm or brain activity evaluation. The core of e-nose is an array of non-specific and cross-sensitive sensors. The array offers advantages, such as increasing e-nose response to a wider range of analytes and improving technology’s accuracy. The sensors most commonly used are metal-oxide semiconducting gas sensors, conducting polymer gas sensors, acoustic sensors or colorimetric sensors [3]. They react to compounds that are mostly volatile organic compounds (VOCs), released from cells and related to metabolism. VOCs are able to promote a change in different sensor’s features (e.g., conductivity, color or oscillation) later translated into a digital visualization called “breathprint”. The curve generated by the VOCs detection is compared to a pattern recognition algorithm to identify the deviation from the standard (i.e., disease free breathprint).

In 1971, Nobel Prize winner Pauling was able to detected hundreds of VOCs in human breath and urine using gas chromatography-mass spectrometry (GC-MS) [4]. Using the same approach, Phillips and colleagues [5] have identified more than 3000 VOCs in exhaled breath. Using GC-MS, VOCs are separated by their mass/charge ratio and identified by a spectral library in the GC software. Starting from that different studies has been conducted to investigate VOCs composition [6,7,8,9]. Even though GC-MS has proved to be an useful methods to identify specific biomarkers, its potentiality is limited due to its features (e.g., complexity, lengthy analysis time, high cost) [10].

On the contrary, non-invasiveness, ease of use both from the physician and patient point of view and rapid response, are the major e-noses’ advantages. Furthermore, the accuracy the instrument can be improved, for instance, by changing array composition enabling multimodal sensing to enhance sensibility and specificity and reduce false positive ratio. Otherwise, breath collection, confounding factors elimination (e.g., drugs assumption, food ingestion) and data analysis can be improved or adapted, thus personalizing the response related to the condition examined. Moreover, considering nanotechnology advancements, we can speculate that in the coming years e-nose technologies will be improve in all its aspect starting from material composition (e.g., surface-to-volume ratios improvement) to sensors performance [11].

E-noses’ characteristics have promoted several clinical trials to evaluate their ability to identify condition-related VOCs discrepancies. This underlines the assumption that different conditions determine the variation of physiological VOCs composition. In vitro experiments have determined e-nose ability to detect VOC patterns in culture medium of several target cells, including lung model cell lines [12,13]. An in vivo approach has confirmed e-noses’ great accuracy to distinguish between healthy subjects and patients with asthma, cystic fibrosis, cirrhotic condition, diabetes, bacterial respiratory tract infection as well as COPD [14].

Moreover, several different e-nose versions have been tested for lung cancer diagnosis and/or progression monitoring (work summarized by Krilaviciute and collagues [15]). Lung cancer is one of the major causes of death worldwide, due to its typical late stage diagnosis. Considering other cancers, such as mammary and colon, the mammography and fecal blood analysis have significantly improved their survival rates. On the contrary, up to now lung cancer has no comparable early diagnosis method. Due to the cancer’s origin (i.e., airways involvement), in recent years interest in exhaled breath analysis through e-nose devices in order to provide accurate results has grown. Accuracy is determined by the combination of sensitivity and specificity, which indicate, respectively, a test’s ability to correctly classify the patient as diseased or disease-free. Those indicators are essential for understanding the effectiveness of the instrument and are indispensable to obtain a reliable early diagnosis and avoid false positives or a missed diagnosis [16]. As demonstrated by Poli and colleagues [17], VOC production and release by lung cancer appear to be an epiphenomenon that accompanies lung cancer development, probably due to the chronic load and burden of VOCs in overall lung tissue.

Even though it is well documented in literature [18] that lung cancer cause a change in VOC signatures and its detectable by e-noses, the missing piece is still the identification of which VOCs are altered in oncological patients. Another major limitation until now has been the lack of a large cohort of patients’ analysis to generate a detailed database, but in the near future, this could be overcome throughout international clinical trial under different respiratory conditions. Nevertheless the advantage linked to robust clinical trial are related also to standardization needed to obtain a translatable approach [19]. This could lead to create a robust algorithm, helping transfer e-noses into clinical practice and expedite diagnosis in several respiratory conditions. This would result in effective screening of large populations with a very high cost-benefit ratio.

Finally, we believe that e-noses satisfy all criteria of a reliable predictive test in terms of simplicity and non-invasiveness, although still more has to be done in terms of scientific validation before they can be considered applicable in clinical practice.
